# Conoccurence of extraskeletal osteosarcoma and undiagnosed Paget disease in a 49-year-old female

**DOI:** 10.1093/jscr/rjae826

**Published:** 2025-01-09

**Authors:** Milena Bogojevska Doksevska, Teodora Todorova, Danica Popovska, Vilijam Velkovski, Tamara Angelovska, Marta Foteva, Slavica Kostadinova Kunovska, Smiljana Bundovska Kocev, Katerina Rebok, Milan Samardziski

**Affiliations:** University Clinic for Orthopedic Diseases, 1000 Skopje, North Macedonia; Faculty of Medicine, Ss. Cyril and Methodius University in Skopje, 1000 Skopje, Republic of North Macedonia; University Clinic for Orthopedic Diseases, 1000 Skopje, North Macedonia; University Clinic for Orthopedic Diseases, 1000 Skopje, North Macedonia; University Clinic for Orthopedic Diseases, 1000 Skopje, North Macedonia; Faculty of Medicine, Ss. Cyril and Methodius University in Skopje, 1000 Skopje, Republic of North Macedonia; Faculty of Medicine, Institute of Pathology, Ss. Cyril and Methodius University, 1000 Skopje, North Macedonia; University Clinic for Orthopedic Diseases, 1000 Skopje, North Macedonia; Faculty of Medicine, Ss. Cyril and Methodius University in Skopje, 1000 Skopje, Republic of North Macedonia; Faculty of Medicine, Institute of Pathology, Ss. Cyril and Methodius University, 1000 Skopje, North Macedonia; University Institute of Radiology, Faculty of Medicine, Ss. Cyril and Methodius University, 1000 Skopje, North Macedonia; Laboratory of Cytology, Histology and Embryology, Faculty of Natural Sciences and Mathematics, Institute of Biology, Ss. Cyril and Methodius University, 1000 Skopje, North Macedonia; University Clinic for Orthopedic Diseases, 1000 Skopje, North Macedonia; Faculty of Medicine, Ss. Cyril and Methodius University in Skopje, 1000 Skopje, Republic of North Macedonia

**Keywords:** extraskeletal osteosarcoma, Paget disease of bone

## Abstract

Extraskeletal osteosarcoma (ESOS) represents a rare soft tissue entity, accounting for ⁓1% of all soft tissue malignancies. It is generally considered to have an even worse prognosis than bone osteosarcoma, therefore detailed investigations and proper treatment are required. ESOSs arising in the subcutaneous tissue are even rarer than the ones positioned in deep tissues, and they are considered to have far better outcomes. We present a case of a 49-year-old patient diagnosed with subcutaneous ESOS and Paget disease of the bone, which is not typical for the patient's age, considering that Paget disease of the bone tends to affect a population above 50 years. The coexistence of these two entities in a single patient and all their features make this case unique, and to the best of our knowledge, this is the first case reported.

## Introduction

Extraskeletal osteosarcoma (ESOS) represents a relatively rare entity, first described by Wilson in 1941 [1]. Consisting only 1% of all soft tissue sarcomas, this tumor tends to appear in older populations, unlike bone osteosarcomas [2]. It frequently occurs in the deep tissues, while subcutaneous location, such as in our case, is extremely rare [3]. As far as our knowledge goes, there are 18 case reports published of subcutaneous ESOSs, but none of them are associated with Paget disease of bone. When it comes to prognosis, the overall survival in patients with ESOS is even worse than in patients with bone osteosarcomas [4]. On the other hand, Paget disease tends to affect the elderly population, it is rarely seen in patients under the age of 50 [5].

What makes our case report unique is the association of these two entities in one patient, ESOS of the lower limb and Paget disease on the iliac wing. To our knowledge, this is the first case report describing these two entities in one patient.

## Case report

**Figure 1:**
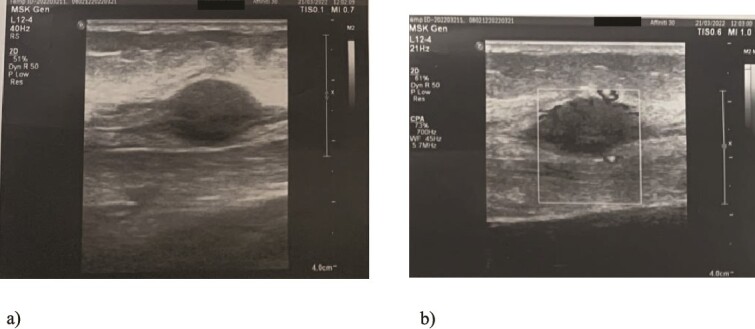
Ultrasound examination of the tumor: Spindle-shaped hypoechogenic and subcutaneously placed mass (a) with marked hypervascularity on color flow mapping (b).

**Figure 2:**
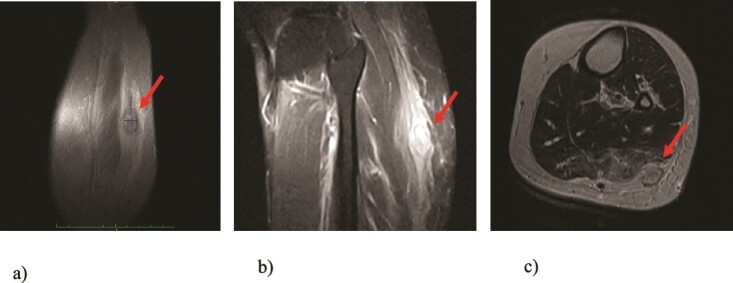
MRI findings of the tumor (arrows): (a) T1 image, coronal view; (b) T2 image, sagittal view; (c) T1 image, axial view revealing presence of a well-defined soft tissue tumor positioned in the subcutaneous fat tissue of the left calf with size of 3 cm and swelling of the surrounding muscles.

**Figure 3:**
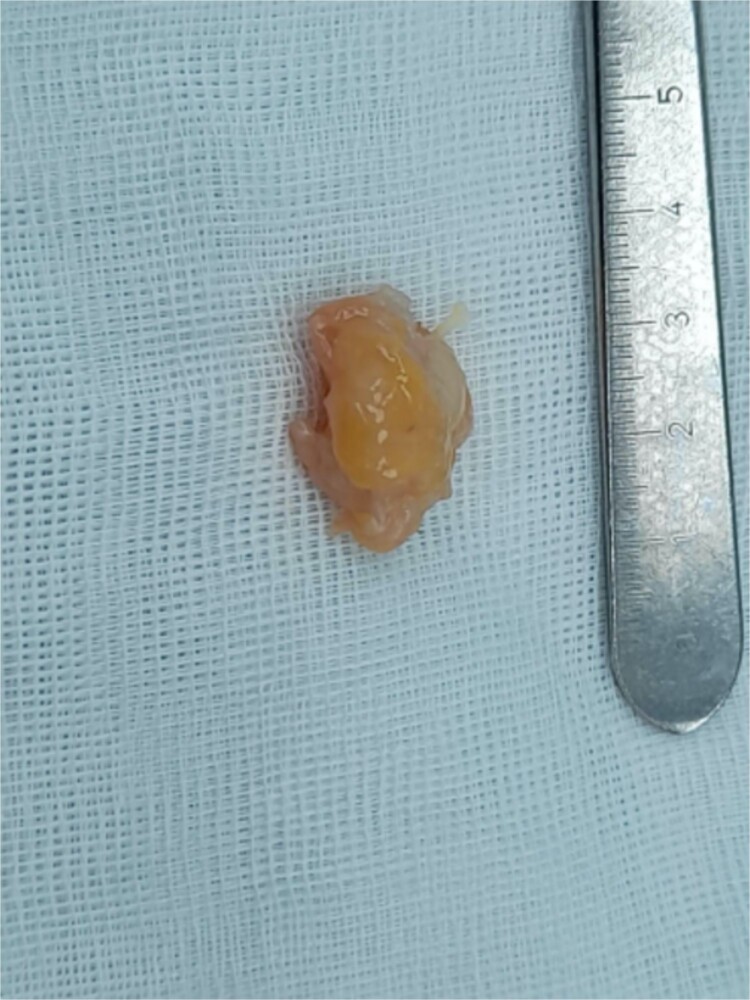
Macroscopic appearance of the specimen.

**Figure 4:**
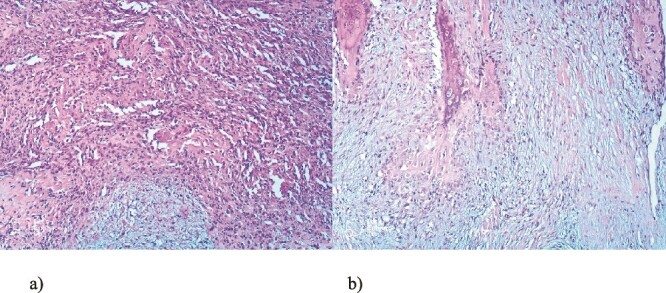
Microscopic appearance of the tumor, He-Eo, ×40.

**Figure 5:**
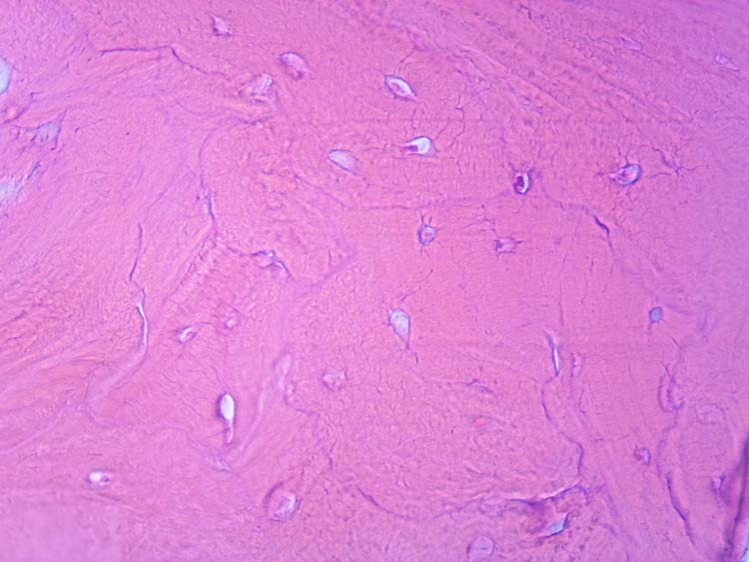
Microscopic appearance, from the biopsy of the iliac wing, showing late (sclerotic) phase of Paget disease, He-Eo, ×200.

We present a case of a 49-year-old, female patient who presented at our clinic complaining of a growing painful mass with a diameter of ⁓3 cm in her left calf. The patient noticed the mass 2 months before the clinical exam and according to the anamnestic data it was growing progressively, becoming more painful, but only when touched, not spontaneously. She was first seen in another clinic, an ultrasound was performed and excision in local anesthesia was suggested. On presentation, clinical and ultrasound examinations were performed. Clinical exam revealed presence of a soft tissue mass on the medial side between the proximal and medial third of the left calf, of ⁓3 cm. It was placed deep in the subdermal area, mobile and not connected to the fascia. The ultrasound showed a heterogeneous, but predominantly hypoechoic mass with increased through transmission and increased vascularization (Fig. 1). Magnetic resonance imaging (MRI) was performed, confirming the presence of a soft tissue tumor in the subcutaneous tissue of the left calf (Fig. 2). Wide resection of the mass under spinal anesthesia was scheduled. Macroscopy of the specimen showed a whiteish soft fragment measuring 2 × 2 × 0.8 cm, microscopically composed of fibroblastic stroma with myxoid degeneration and immature osteoid embedded in between atypical osteoblasts with inconspicuous mitotic activity (Fig. 3). Foci of chondroid matrix with atypical chondrocytes were present. Necrotic and calcified bony trabeculae with osteoblastic rimming and adipose tissue with skeletal muscle were present on the periphery. The proliferative index, Ki67 was ⁓5%, thereupon a diagnosis of low-grade ESOS was made (Fig. 4). The regular staging follow-up was performed with chest and abdominal computed tomography (CT). No signs of metastatic disease were detected. The bone scan with Tc99m showed an increased uptake on the left iliac bone, highly suspected of Paget disease. Another surgery was scheduled. This time, re-resection procedure to achieve negative margins, as well as a biopsy of the iliac wing was performed. Negative margins were accomplished with the re-resection procedure. Grossly, the iliac bone biopsy was composed of five bony fragments measuring 0.5–1.5 cm. Microscopy showed sclerotic and irregularly shaped anastomosing, lamellar bony trabeculae with focal irregular calcification. The trabeculae contain osteocytes without atypia, rimmed with reactive osteoblasts, as well as multinucleated osteoclast giant cells. Granulation tissue with focal collagen deposition was present focally in between the bone trabeculae. Focal bone marrow was present. The microscopic analysis was consistent with the late (sclerotic) phase of Paget disease of bone (Fig. 5). On the last follow-up exam, 2 years after the surgery there is no evidence of local recurrence.

## Discussion

The occurrence of soft tissue tumors adjacent to Paget disease is not common. It is considered that this is mainly due to unmineralized osteoid [5]. Genetic changes most commonly associated with osteosarcoma are the mutations of the p53 – tumor suppressor gene and retinoblastoma gene, RB1. Its association with Paget disease is detected on the chromosome 18q [6, 7].

Our literature research did not reveal any reports of the association of ESOS and Paget disease of bone.

ESOS represents a malignant mesenchymal tumor, characterized by the presence of a slow-growing mass, with or without minimal bone attachment. In around 12.5% of the patients previous history of trauma is present. The most common localization is the thigh, but it can appear anywhere in the body. Around 4%–13% of the reported ESOSs occur secondary as a result of radiation therapy [8, 9].

Regarding the differential diagnosis, lesions such as myositis ossificans after a previous trauma event and synovial sarcoma should be taken into consideration, which can be easily excluded after detailed diagnostic investigations, which will confirm the presence of mineralized soft tissue mass [9].

Standard treatment consists of wide resection or if required, amputation. Available studies suggest that there is no significant difference in the overall survival between the two surgical treatments. An adjuvant chemotherapy regimen is indicated in patients with high-grade ESOSs, while radiotherapy is reserved for palliative purposes or in cases of marginal surgical resection [10, 11].

Overall it is proved that the presence of Paget disease of the bone is associated with an increased risk for osteosarcoma development. In our case, a biopsy of the left iliac crest confirmed the diagnosis of Paget disease of bone, and the patient was started on bisphosphonates therapy.

It is important to report such unusual cases and to keep in mind that their simultaneous occurrence is possible. Further genomic and molecular studies are needed to discover the possible underlying mechanism of their occurrence.
